# Continuous Electronic Fetal Monitoring during Labor: A Critique and a Reply to Contemporary Proponents

**DOI:** 10.1055/s-0038-1632404

**Published:** 2018-03-07

**Authors:** Thomas P. Sartwelle, James C. Johnston

**Affiliations:** 1 Deans and Lyons, LLP, Houston, Texas; 2 Global Neurology Consultants, USA and Auckland, New Zealand

**Keywords:** electronic fetal monitoring, cerebral palsy, medical ethics, journal of child neurology, Dr. Max Wiznitzer, Dr. Marc Patterson

## Abstract

A half century after continuous electronic fetal monitoring (EFM) became the omnipresent standard of care for the vast majority of labors in the developed countries, and the cornerstone for cerebral palsy litigation, EFM advocates still do not have any scientific evidence justifying EFM use in most labors or courtrooms. Yet, these EFM proponents continue rationalizing the procedure with a rhetorical fog of meaningless words, misleading statistics, archaic concepts, and a complete disregard for medical ethics. This article illustrates the current state of affairs by providing an evidence-based review penetrating the rhetorical fog of a prototypical EFM advocate.

## Prologue

Four centuries ago, the Catholic Church declared heliocentrism to be heretical, banned all heliocentric books, and ordered Galileo to refrain from holding, teaching, or defending heliocentric ideas. A similar effort is taking place today. It seeks to suppress debate over birth caregivers' half-century misguided use of electronic fetal monitoring (EFM) and cesarean sections (C-sections) to allegedly prevent cerebral palsy (CP). 1 2 The theory—EFM predicts CP and just-in-time C-sections and prevents CP 1 2 —has caused more harm than good to mothers and babies 3 4 5 and has resulted in decades-long, worldwide EFM-CP litigation that has reached a crisis of near epidemic proportions, 6 costing tens of millions annually. 1 6 7

The following article has its genesis in exposing an EFM-CP litigation apologist—Dr. Max Wiznitzer—who defended the current EFM-CP status quo in an ambush “editorial” 8 he authored by special invitation from the current editor Dr. Marc Patterson and senior editorial staff of the Journal of Child Neurology (JCN). This special invitation and the editorial exposed Dr. Patterson's and editorial staff's bias for the CP-EFM status quo as well as their efforts to muffle EFM-CP critics.

In 2014, JCN, led by a different editor, published the current authors' article— *Cerebral Palsy Litigation: Change Course or Abandoned Ship* 9 —highly critical of EFM and criticizing EFM-CP litigation as a deceitful, money wasting sham on physicians and the public. After *Abandon Ship's* publication, Dr. Patterson took over JCN. He surreptitiously engaged Wiznitzer—a JCN Editorial Board member and a child neurologist—to write the “editorial” as a reply to *Abandon Ship.* Dr. Patterson published the editorial almost *2 years* after Abandon Ship's publication. These authors received no notice of either the preparation of the editorial or its publication. The “editorial” hid from JCN readers the fact that Wiznitzer was a JCN editorial Board member and career expert witness, and the editor also allowed Wiznitzer to engage in unprovoked, spurious personal attacks on these authors. The editorial, as will be seen from this article, is so lacking in scholarship and references that it is virtually certain that it was not peer-reviewed.

These authors replied to the “editorial” by submitting the foregoing article to JCN in accordance with the usual instructions for authors. In reply, the authors received written confirmation that the article “is presently being given full consideration for publication in the Journal of Child Neurology.” JCN, as does any legitimate medical journal, promises “ALL submissions (including invited material) are subjected to peer review.”

But Dr. Patterson, the same JCN editor who surreptitiously planned and executed the ambush “editorial,” unilaterally refused to submit the manuscript to peer review despite informing the authors for months that the manuscript was undergoing routine peer review. Finally, Dr. Patterson simply e-mailed a rejection 4 months after submission, stating that all discussion of the topic “is now closed.”

The hallmark of real science is submitting existing paradigms to the crucible of vigorous debate. If CP-EFM use in labor and litigation can be justified as science, then perhaps EFM use will continue. But the debate deserves open discussion not suppression by partisan apologists.

The following article refutes Wiznitzer's “editorial” and defends these authors' call for the abolition of EFM in labor as well as in CP-EFM litigation. This article, contrary to the editorial, relies on current scientific research and facts to support these arguments. The question is: can proponents of continuing the current EFM paradigm for labor and litigation do the same?

## The Fog of Rhetoric

A half century after continuous EFM became the omnipresent standard of care for the vast majority of labors in the industrialized world, 1 3 9 and became the cornerstone for EFM-CP litigation, 1 3 6 9 EFM proponents still have no valid scientific evidence justifying EFM use in most labors or in CP litigation. 1 2 3 4 5 6 7 9 10 11 12 Nevertheless, EFM proponents continue rationalizing EFM use in labor and courtrooms with a rhetorical fog of misleading statistics, meaningless words, and reliance on concepts as archaic as the miasma theory of diseases.

Emblematic of this rhetorical fog is a recent “editorial” defending past and current EFM use as science, championing its litigation use to unjustly convict birth caregivers of causing CP and rationalizing continued EFM-induced unnecessary C-sections causing harm to mothers and babies, with the half-century-old bromide that “more research is needed.” 8

This article will penetrate the rhetorical fog of this latest EFM apologist and others advocating continued EFM use despite the overwhelming evidence of its uselessness in labor and its junk science status in litigation.

## Motivation

A cursory review of Wiznitzer's editorial 8 makes it seem as if it was prompted by his altruistic, academic desire to defend EFM from the courtroom junk science label. In reality, the junk science label is of special concern to him because, as disclosed in his conflict of interest statement, he testifies for money in encephalopathy lawsuits. 8 What he failed to disclose, however, was his 2015 testimony under oath that he spends 4 to 5 hours per week in paid litigation. 13 Almost 300 hours per year is a significant source of income, especially when he has testified since at least 1998. 14 Wiznitzer's real motive, therefore, would seem to be protecting his EFM-CP litigation testimony from attack as junk science and possibly being struck as a witness in forthcoming trials. 15

Wiznitzer's justification for current EFM use in virtually every pregnancy and in CP litigation—the current status quo—is summarized in these confected arguments: EFM is efficacious because it “is widely used in the obstetrics community”; EFM has been “investigated with resultant publications”; CP due to hypoxic–ischemic encephalopathy (HIE) occurs in 1 to 4 per 10,000 live births; animal and human studies show correlation between EFM changes and fetal acidemia; clinical trials between EFM and intermittent auscultation (IA) to detect CP were underpowered and could not detect differences in that outcome; EFM has a biological basis supporting its continued use; and intensive EFM training in a few limited studies resulted in improved neonatal outcomes. 8

These arguments will be examined against the background of EFM-CP research and experience.

## Is Electronic Fetal Monitoring Junk Science?

Five decades of overwhelming evidence prove that EFM is not only unscientific 1 3 9 10 11 12 but also has driven the C-section rate to unprecedented levels—one-third of babies in the United States and a quarter in the United Kingdom, 1 and Australia, 16 delivered by C-section—despite evidence that EFM and cesareans have not altered in the least the rate of CP, 1 2 3 4 5 9 10 11 12 which was the primary aim of EFM, 1 2 3 4 5 nor has it altered the rates of perinatal death, 1 2 neonatal death, 1 2 intrapartum stillbirth, 1 2 low or very low Apgar scores, need for special neonatal care, 1 2 or the rate of neonatal encephalopathy. 1 2 17

While EFM failed as a medical appurtenance to birth, EFM use did produce the ongoing worldwide CP litigation conflagration 1 6 9 with the counterfeit foundation that EFM predicts and prevents CP, and EFM-inspired just-in-time C-sections save babies from a lifetime of CP and other neurologic devastation. 1 3 4 5 6 7 10 11 12 18 CP litigation, in turn, inspired physicians to compromise their ethical principles, using EFM without informed consent as protection from CP lawsuits despite the evidence that EFM has caused more harm than good. 9 11 18 19

## Widely Used in the Obstetrics Community

Wiznitzer's first argument is known by many names—argumentum ad populum, bandwagon fallacy, appeal to popularity, consensus fallacy—but is a poor substitute for medical evidence. One may as well argue that since millions of people worldwide still smoke, smoking must be healthy despite the contrary medical evidence. And not so long ago, the medical community's conventional wisdom was the belief in the miasma theory and the imbalance of the body's “four humours” as the consensus cause of disease. When challenged by Semmelweis' simple hand washing to prevent puerperal fever, the collective medical community rejected the idea outright for almost a half century.

There are hundreds of other past and current examples of widely held medical community fallacies. 20 21 22 In this age of evidence-based medicine, a modality like EFM should certainly not rest on a belief that it is efficacious merely because it “is widely used in the obstetrics community,” 8 especially when multiple lives are at risk as in labor and delivery.

## Electronic Fetal Monitoring Investigated and Published

Wiznitzer argues that EFM is science because EFM has been investigated and the investigations have been published. 8 But Wiznitzer asks readers to have faith and trust in his statement on his say-so alone because he provides no citations to any of the published “investigations” he claims exist. A significant omission in the milieu of today's evidence-based medicine, reminiscent of days gone by when noted American zoologist Theophilus Painter in 1923 declared that humans had 24 pairs of chromosomes. Until 1955, this erroneous concept was consensus scientific opinion based solely on Painter's unquestioned authority. 23 Wiznitzer fails to cite articles supporting EFM as science because there are no contemporary articles supporting that statement.

EFM was invented in the hope of identifying fetal asphyxia, which was thought to be the cause of CP. 1 3 10 EFM inventors, with significant undisclosed conflicts of interest, 11 24 25 relied on anecdotes rather than clinical trials to “prove” EFM efficacy. 1 7 9 10 24 25 In 1975, a decade after EFM's first clinical use but before EFM was subjected to even one clinical trial, advocates contended that EFM alone would reduce by half intrapartum deaths, mental retardation, and CP. 26

But even as EFM was becoming the standard of care despite a lack of clinical trials, more scientifically motivated practitioners were using science to test EFM's underlying theory—fetal heart rate patterns predict fetal distress (asphyxia) and acidosis. 7 24 25 Benson et al, studying 25,000 labors, concluded there was no single reliable fetal heart rate indicator of fetal distress, 27 whereas others demonstrated that most fetal heart rate patterns deemed abnormal were associated with normal cord pH, and even the most dramatic patterns resulted in very few neonates born acidotic. 4 24 25 The obstetrical community ignored these studies, purposely using unproven EFM technology in an age when medical technology seemed to have bypassed obstetrics in favor of every other medical specialty. 9 11 18 24 25

For the next half century, the scientific and medical evidence related to CP and EFM accumulated at a rapid pace. At the same time, EFM use increased to 85% of all labors in the United States and similar numbers in other industrialized countries. 7 9 12 28 The rate of C-sections worldwide also rose substantially. 1 Today, C-sections are used to deliver one-third of babies born in the United States and similar percentages in other countries, 1 4 16 28 rates that exceed the rate needed to minimize morbidity and mortality. 29

But like an O. Henry short story, the EFM saga has a plot twist and a surprise ending. The accumulated CP-EFM evidence did not prove asphyxia as a prominent CP cause or that EFM was efficacious. Rather, the evidence proved exactly what Benson 27 and others said about EFM in the beginning: birth asphyxia was a rare cause of CP; 1 2 3 4 5 6 7 9 10 11 12 17 18 19 EFM as a predictor of CP was a myth; 1 2 3 4 5 6 7 9 10 11 12 17 18 19 EFM inspired C-sections caused more harm than good; 4 5 9 and the rate of CP, despite EFM and C-sections, was unchanged, as were rates of perinatal death, intrapartum stillbirth, neonatal death, low or very low Apgar scores, need for special neonatal care, and neonatal deaths. 1 2 3 4 5 7 9 10 11 12

The more incredible part of this O. Henry ending is the fact that this accumulated evidence is virtually unchallenged by any substantial contemporary medical or scientific evidence. So overwhelming is the evidence that a prestigious group of maternal–fetal medicine specialists have advocated that it is time to start over with EFM and establish common language, standard interpretation, and reasonable management principles and guidelines. 30 The obstetrical societies of the Australia, Canada, New Zealand, and the United States, assessing the CP-EFM evidence, agree that EFM provides no long-term benefit for children. 17 Since 2001, Cochrane Database of Systematic Reviews has reviewed EFM four times, each time concluding that there is no difference between EFM monitoring and IA (with one parameter discussed later) except that the C-section rate with EFM is substantially higher. 2 The United States Preventive Services Task Force gave EFM a D grade, the worst that can be given, concluding that there was no evidence of EFM benefit and substantial evidence of harm. 4

So Wiznitzer's statement that EFM has been investigated and published is correct. His implication that the investigation resulted in a finding that EFM is scientific is so wrong as to be intentionally dishonest.

## Cerebral Palsy due to Hypoxic Ischemic Encephalopathy

Wiznitzer begins discussing HIE with a demonstrably false premise: “In order to determine whether studies of the use of electronic fetal monitoring *in LESSENING the cerebral palsy rate have reached valid conclusions,* an understanding of the epidemiology of cerebral palsy is needed.” 8 [emphasis added]. There is no published study concluding that EFM has ever *lessened* the CP rate, just the opposite. EFM's lack of effectiveness in preventing CP is consistent: Since 1970 when EFM became the default standard of care, and C-sections the misguided rescue procedure for supposed fetal distress-asphyxia, the world rate of CP has remained unchanged. 1 5 6 7 9 12

From this false premise, Wiznitzer cites statistics from *Neonatal Encephalopathy 2014 (NE 2014)* 17 ending with a calculation that 10 to 20% of CP is due to HIE, a rate of 1 to 4 per 10,000 live births. 8 This calculation, however, seems rather bizarre because it does not lead to any relevant conclusion.

Perhaps more telling, the source for the statistics, *NE 2014,* first counsels the readers not to use the HIE terminology because “neither hypoxia nor ischemia can be assumed to have been the unique initiating mechanism” of CP. 17 Second, *NE 2014* warns the reader that the statistics are based on studies where no two authors used the same neonatal encephalopathy definition or criteria, thereby causing significant terminological confusion and misuse of the various statistics. 17

One wonders why Wiznitzer uses stale, misleading, outdated data and terminology when several contemporary articles by highly visible, respected, original CP researchers—Jonas Ellenberg, Karin Nelson, Nadia Badawi and Alastair MacLennan, to name just a few—have analyzed contemporary data and CP causes. 7 10 31

Ellenberg and Nelson published the first CP–asphyxia analysis in a 1986 landmark article, concluding that the proportion of CP reasonably attributable to birth asphyxia was less than 10%. 32 Revisiting the issue in 2013, they analyzed the literature published since their 1986 article. Their conclusion: “The current data do not support the belief, widely held in the medical and legal communities, that birth asphyxia can be recognized reliably and specifically on the basis of clinical signs such as aberrant fetal heart rate patterns, Apgar scores, respiratory depression, neonatal seizures, or acidosis, or that most CP is due to birth asphyxia.” 31

Badawi, an internationally recognized CP researcher, and among the first to conduct reliable research on CP causes, also analyzed the causal pathways to CP, observing that most term infants with CP seem normal at birth. Only 25% of term infants in the Western Australia Newborn Encephalopathy study who develop CP had neonatal encephalopathy, and 29% had identifiable intrapartum risk factors, of whom 24% had both antenatal and intrapartum risk factors and only 5% had solely intrapartum risk factors. 7

MacLennan, also an internationally recognized Australian CP researcher and prolific author, in a 2015 article that should have been available to Wiznitzer, concluded that the origin of most CP is prior to labor and is due to a host of causes and pathways including up to 31% due to genetic variants. 10 33

It is indeed bizarre that Wiznitzer never mentions these international icons of CP research whose research has dominated the medical journals for decades.

## Electronic Fetal Monitoring and Acidemia

Wiznitzer apparently believes that EFM is scientific because “animal and human studies showed a correlation between EFM changes and fetal acidemia.” 8 Again, with no citation to support the statement, the reader is back to having to trust the writer's veracity. And in this case, such trust would be highly misplaced. Early in EFM's life, it was well known that most EFM patterns thought to be abnormal were associated with normal cord pH, and even with dramatic patterns, a very small proportion of neonates were born acidotic. 4 And despite whatever animal and human studies Wiznitzer may have read, contemporary efforts to correlate EFM patterns with neonatal acidemia have failed. 34 35 36 37 38

## Previous Electronic Fetal Monitoring Studies Underpowered

The underpowered studies argument is a familiar one. Wiznitzer seems to have been inspired in this argument by the 2015 International Federation of Gynecology and Obstetrics (FIGO) Consensus Guidelines on Intrapartum Fetal Monitoring, 39 which made the exact same argument: EFM and IA comparative studies, undertaken in the 1970s, 1980s, and early 1990s, were underpowered to detect differences in CP and other major outcomes, and therefore the results are difficult to relate to current practice, 8 the thought being that current-day physicians should ignore these studies. FIGO stated it plainly: most experts believe that EFM should continue to be used. 39 The number representing “most” was unstated.

This argument is simply a canard, an easily destroyed straw man, made so that “most experts” can continue EFM use exactly as they have from the beginning. The previous studies may be underpowered, but is that a sufficient rationalization to continue EFM use? The question unanswered by FIGO and Wiznitzer is: where are the studies justifying continued EFM use? In this day of evidenced-based medicine, the argument that most experts favor its use as a substitute for scientific proof is pious, duplicitous, sanctimonious, and hypocritical. Wiznitzer and FIGO need to join the Choosing Wisely Canada initiative to find what medical “activities that, based on evidence, we should not be doing and then try not doing those things. By doing less, not more, we will spare patients harm and can free up resources for what is really helpful.” 40

## Electronic Fetal Monitoring's Biological Basis

Wiznitzer claims that EFM has a biological basis and, when used as part of an ongoing quality improvement program, results in improved neonatal outcomes. 8 Again, however, there are no citations supporting the biological basis statement (the quality improvement statement is refuted in the next section). It is difficult in the extreme to determine what Wiznitzer relies on for this assertion since EFM's biological basis has been questioned from the beginning and throughout its existence and proven false. 1 2 3 4 7 9 10 11 12 15 16 17 18 19 24 25 27 30 31 32 More recently, it has been shown that fetal heart rate decelerations, the heart of EFM orthodoxy, are commonly associated with unthreading peripheral chemoreflex rather than fetal head or cord compression, the mainstay EFM dogma for five decades. 41

## Electronic Fetal Monitoring Quality Improvement Programs

Wiznitzer cites four articles 8 (2006, 2009, 2014, 2015) involving intensive EFM pattern recognition and communication training programs resulting in what were said to be improved neonatal outcomes, albeit the improvements were temporary and went away if the intense training stopped. The so-called improvements were also questionable as the criteria for what were called “improved results” are subjective and elusive.

The most important and recent of these studies recognized several shortcomings for this type of study, not the least of which was that the improvements gained were for short-term neonatal outcomes. These authors cautioned that there was little correlation between the outcomes examined, and neonatal encephalopathy and other long-term adverse sequelae of labor. 42

Despite these shortcomings, Wiznitzer contends that these studies combined with EFM's biological basis prove that EFM is not junk science. Thus, Wiznitzer claims that only “more research is needed to define the use of electronic monitoring in appropriate target populations, in determination of neonatal outcome, in utilization with other measures of fetal functioning, and in quality improvement and education programs.” 8 This passage is the typical rhetorical fog that EFM proponents resort to because they have no evidence. The sentence has no meaning. It could be used in a dictionary to illustrate words such as obtuse, nonsense, nonsequitur, inane, vacuous, and orthogonal. It could easily have been written by Lewis Carroll as part of Alice's dialog with any of the characters in *Alice's Adventures in Wonderland.*

The EFM “more research is needed” canard has been used for most of EFM's clinical life to justify continued EFM use despite the known harm it caused to mothers and babies. 9 11 18 25 28 In view of the known EFM harms, it is time for obstetrics to recognize that EFM's apotheosis was the day of its first clinical use. Fifty years of EFM research has only brought EFM to the point where all but EFM zealots recognize it is time to start over. 1 4 9 10 19 30

In fact, many in the obstetrical world have recognized EFM's harms, contrary to Wiznitzer and his ilk, and tried to reduce its impact on at least low-risk pregnancies. In the forefront of this effort has been United Kingdom's National Institute for Health and Care Excellence (NICE). Since 2001, NICE has slowly reduced EFM use and has for many years recommended to not use EFM in normal pregnancies. 43 Recently, ACOG made similar recommendations. 44 Recognizing that EFM was introduced as a substitute for IA but that EFM has not reduced perinatal death or CP, but has substantially increased the numbers of C-sections and instrumented vaginal deliveries, ACOG now recommends that women with low-risk pregnancies be given an informed choice between IA and EFM. 44

## Electronic Fetal Monitoring Reduces Seizures?

The only EFM red herring Wiznitzer did not use as a rationalization for continued EFM use was that EFM use reduces neonatal seizures. It is true that the EFM versus IA clinical trials that EFM proponents claim were underpowered and therefore useless have shown a reduction in neonatal seizures. 2 These same trials have also demonstrated no significant differences in CP, infant mortality, or other standard measures of neonatal well-being but demonstrated significant increases in caesarian sections and instrumented deliveries. 2

But the reduction in seizures is an ephemeral “benefit” because there have never been any long-term follow-up data assessing the importance of this reduction. 2 Labeling seizure reduction as an EFM “benefit,” therefore, is simply grasping at nonexistent straws. Importantly, assuming a seizure risk of approximately 3 per 1,000, it is estimated that 667 women must be subjected to continuous EFM monitoring to prevent one seizure. And of the 667 women so monitored, 15 would have C-sections. 2

## Conclusion

Fifty years of EFM use has resulted in more harm than good to mothers and their children. When it comes to labeling a medical modality “junk science,” is there any criteria more significant? Medical research frequently reveals some long-held belief to be obsolete or even harmful, and it is discarded. EFM has been harmful for almost its entire existence, yet EFM apologists like Wiznitzer and others continue to obfuscate the truth with their rhetorical fog of stale facts and willfully ignore EFM harms, insisting that EFM is a viable medical tool. Why?

The answer to this question is elusive at best and lays somewhere in the collective unconscious mind of EFM zealots. As Churchill said of Russia, it is a riddle wrapped in a mystery inside an enigma.
